# Restorative Management of Severe Localized Tooth Wear Using a Supraoccluding Appliance: A 5-Year Follow-Up

**DOI:** 10.1155/2018/9864782

**Published:** 2018-05-27

**Authors:** Tsz Leung Wong, Michael George Botelho

**Affiliations:** Prosthodontics, Faculty of Dentistry, The University of Hong Kong, Sai Ying Pun, Hong Kong

## Abstract

This case report illustrates a novel conservative restorative management of a patient with bulimia nervosa who presented with severe localized upper palatal tooth wear and an anterior reverse overjet. This was achieved by using a localized bite raising or supraoccluding appliance, cemented on the lingual side of the lower anterior teeth to create interocclusal space, obviating the need for tooth reduction of the eroded upper palatal and incisal tooth surfaces. Surgical crown lengthening was performed to create a better aesthetic gingival architecture. All-ceramic restorations were provided on the upper anterior teeth to restore the tooth surface loss and provide a positive overbite and overjet. There was no complication or other observable biological change detected at the 5-year follow-up. The use of an appliance applying the supraoccluding technique, or Dahl concept, is a safe, conservative, and useful treatment option in the management of localized tooth wear.

## 1. Introduction

Restoration of the localized worn dentition, especially in the aesthetic zone, is often challenging, as the localized tooth loss can be accompanied by compensatory tooth eruption and alveolar bone growth such that the worn surface will maintain contact with the opposing dentition [1, 2]. This may lead to an irregular gingival contour or level and may result in compromised aesthetics. Also, tooth preparation of such worn teeth to create interocclusal space for the planned restorations may endanger pulp vitality. In addition, tooth wear from erosion may reduce the crown height which in turn reduces the resistance and retention form which may warrant surgical crown lengthening to overcome this. Elective devitalization and the use of postcore crowns may solve the retention problem; however, this may significantly affect the long-term prognosis of these teeth. Orthodontic intrusion can be considered to create space; however, treatment cost, time duration, or patient's preference may exclude this treatment. Increasing the occlusal vertical dimension (OVD) in a full mouth rehabilitation is an alternative to create the necessary space. However, this is usually considered when the tooth surface loss is generalized and the whole arch needs restoring which makes this cost prohibitive to many patients. Space for restoration can also be created by occlusal adjustment of the anteroposterior discrepancy between the centric occlusion and centric relation. However, such discrepancies are not always present and can still involve multiple restorations.

An alternative approach described by Dahl is a technique where interocclusal space is created using an anterior bite platform [3, 4]. The platform was designed to disclude the posterior teeth with subsequent reestablishment of the posterior occlusion from a combination of intrusion of the anterior teeth and overeruption of the posterior teeth [5, 6]. This takes an average of about 6 months to occur [7, 8]. This technique was shown to be safe and can be a conservative and useful option in the management of the localized worn dentition [7–9]. The original Dahl appliance was a removable one covering the palatal surface of the upper anterior teeth [3, 4]. However, this is not applicable to patients with reverse overjet, as extension of the metal coverage beyond the incisolabial and in supraocclusion with the opposing would create an aesthetic problem and a high dislodging force when biting. In this case report, a novel appliance applying the Dahl concept was designed in the localized management of worn anterior teeth. There appears to have been no previous report of the use of a similar lower bite platform in the creation of interocclusal space.

## 2. Case Presentation

### 2.1. History and Chief Complaint

A 33-year-old Chinese female presented with concerns about the appearance of her anterior teeth and was referred by a private dental practitioner to the Prince Philip Dental Hospital (PPDH) for aesthetic management of her upper worn anterior teeth. History revealed that she had endodontic treatment on her two upper central incisors because of pain. She also had some tenderness on her lower right molar when chewing. She reported gastric regurgitation after eating but denied self-induced purging at that time.

### 2.2. Clinical Findings

**Figure 1:**
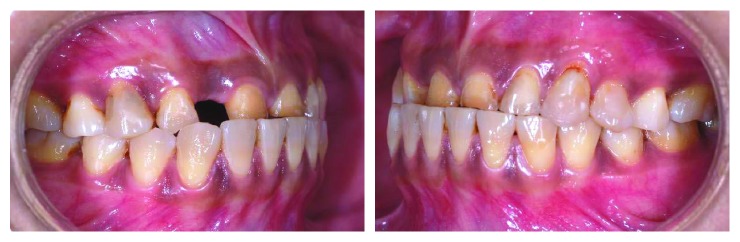
Preoperative lateral views with teeth in centric occlusion.

**Figure 2:**
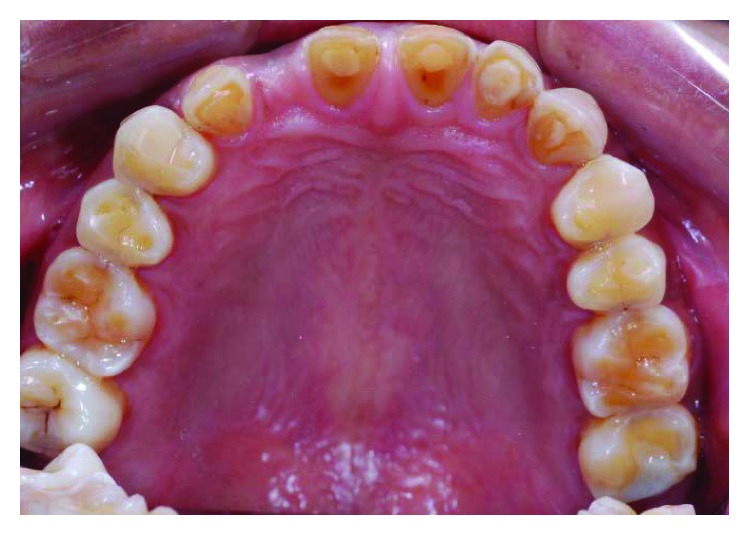
Preoperative upper occlusal view.

**Figure 3:**
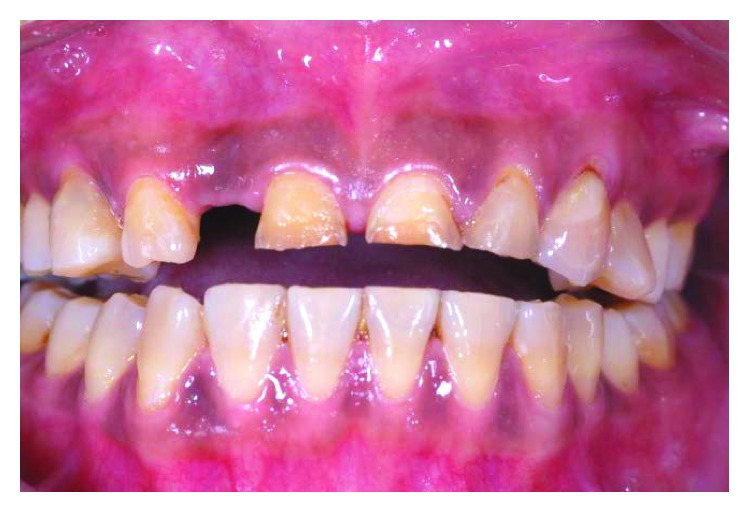
Preoperative frontal view with teeth apart.

**Figure 4:**
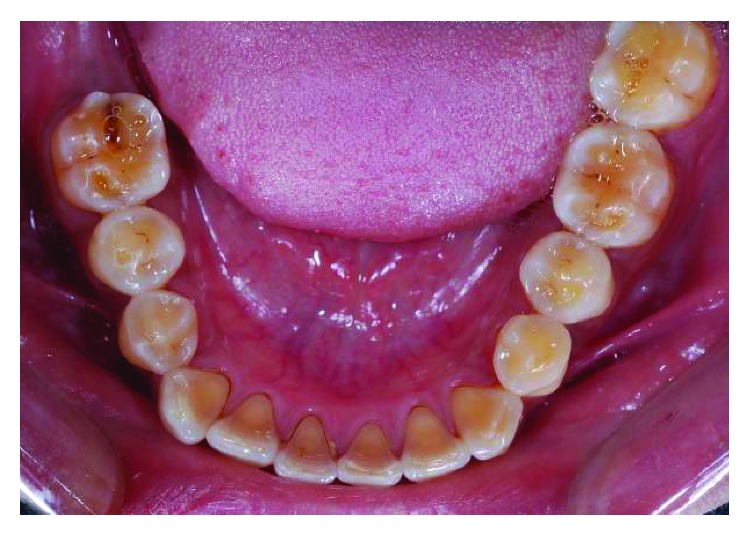
Preoperative lower occlusal view.

**Figure 5:**
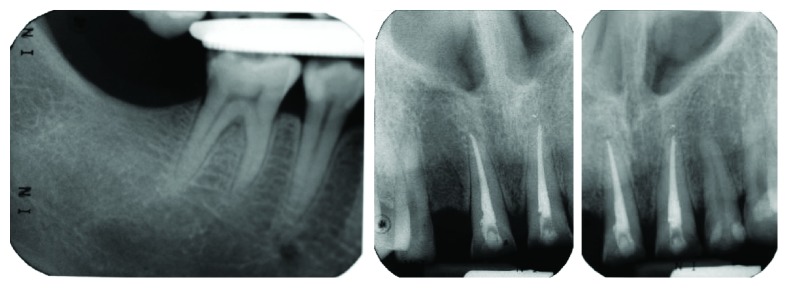
Preoperative periapical of 46 and upper anterior teeth showing no periapical radiolucency with good alveolar bone level.

This patient presented with competent lips, average lip line, normal TMJ's, and class III incisor relation (Figure 1) on a skeletal class 3 base. The patient had good oral hygiene and a healthy periodontal condition. Tooth 13 was missing and 12 had drifted to the 13 position (Figure 2). There was severe erosive tooth surface loss on the palatal and incisal surfaces of teeth 11, 21, and 22 with exposure of dentine over the whole palatal surface. The crown height of the two upper central incisors was reduced to 5 mm. This gave rise to a reduced visible crown height and a reverse smile line (Figure 3). The gingival level of the upper central incisors was level with the lateral incisors and below that of the canine teeth. A crack line was detected at 46 distoocclusal area (Figure 4) with no periapical radiolucency (Figure 5), and a diagnosis of crack tooth syndrome was suspected. Teeth 12, 22, 23, and 46 were judged vital with the use of electric pulp testing.

### 2.3. Diagnosis and Treatment Plan

This patient was advised to seek medical consultation and management concerning her gastric regurgitation. She received conservative medical counseling for approximately one year, and her gastrointestinal disturbance improved. To prevent further symptoms and crack propagation, an orthodontic band was cemented on 46 after which no pain during chewing was reported. Upper and lower impressions were recorded using irreversible hydrocolloid (Aroma Fine Plus, GC Corporation, Tokyo, Japan). A facebow record was taken, and the casts were hand articulated and mounted in maximum intercuspal position. After analysis of the study models, it was decided to perform localized “intrusion” of the anterior teeth using a lower supraoccluding appliance and to build up the correct contour of upper incisors to facilitate the restorative rehabilitation. It was determined that an increase in the OVD by 1.5 mm would provide sufficient space for restoration. To control the anticipated increased OVD for the composite build-up, a silicone putty (Exafine Putty, GC Corporation, Tokyo, Japan) jaw record was performed on the posterior teeth of the articulated study models as a reference jig.

**Figure 6:**
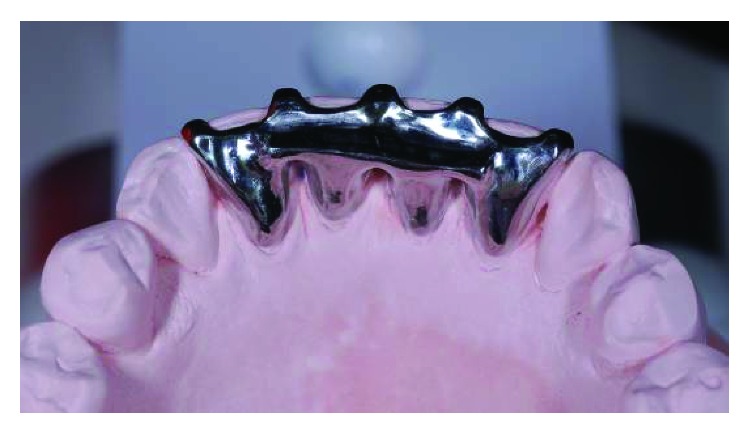
Lingual view of the bite platform on working model.

**Figure 7:**
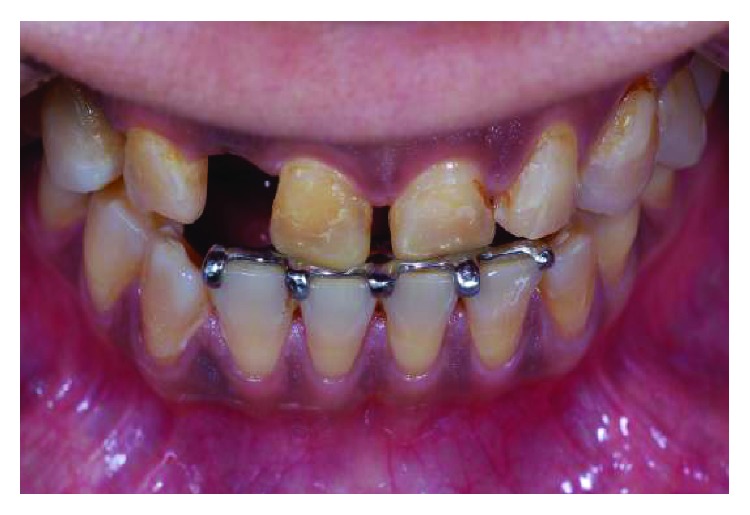
Stable occlusion of numbers 11 and 21 on the lingual bite platform.

**Figure 8:**
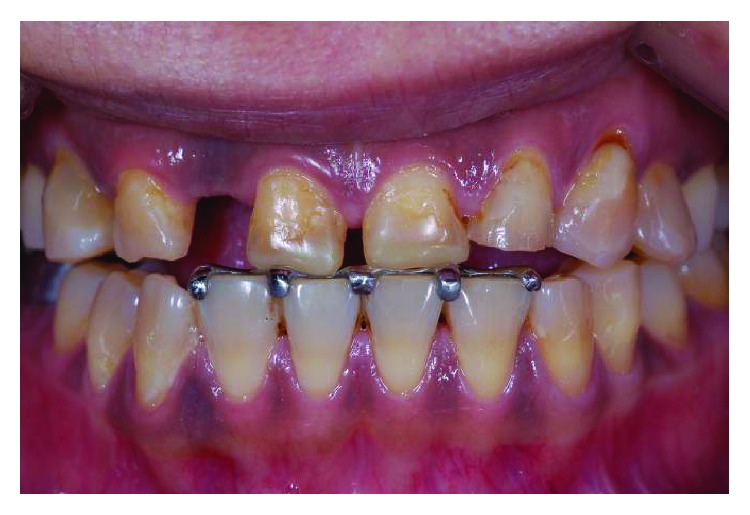
The appliance in situ with composite build-up in supraocclusion on numbers 11 and 21 incisal to separate all other teeth by 1.5 mm. The design of using incisal hooks on the lower teeth helps improve the resistance to dislodgement of the appliance.

The supraoccluding appliance was waxed up on the lingual surface of the lower incisors with a bite platform designed to load the opposing incisor axially. Incisal hooks were included to give the appliance resistance form. This was then cast in cobalt-chromium (CoCr) (Remanium® GM 800+, DENTAURUM GmbH & Co., Ispringen, Germany) (Figure 6). The appliance was then cemented on lower incisors with glass ionomer cement (Ketac Cem, ESPE-Premier Sales Corp., Norristown, PA, USA). At the same visit, composite resin (Aeliteflo™, Bisco Inc., Schaumburg, IL, U.S.A) was directly added to restore the incisal edges of 11 and 21 and facilitate occlusion on the opposing bite platform at the increased OVD of 1.5 mm (Figures 7 and 8) using a silicone putty jaw record as a jig.

The patient was informed of possible transitory problems with this supraoccluding technique, including thermal sensitivity; difficulty in eating, speaking, or sleeping; and temporomandibular joint (TMJ) pain [10]. This patient was reviewed at one week and then at monthly intervals. She reported only some reduced chewing efficiency at the first review and no other symptoms. In order to determine whether tooth movement had occurred, an interocclusal record using Protemp (Protemp II, ESPE, Seefeld, Germany) was taken and it was then inserted at the next review appointment. If tooth movement had occurred, there would be interocclusal separation of the upper anterior teeth with the bite platform of the appliance.

**Figure 9:**
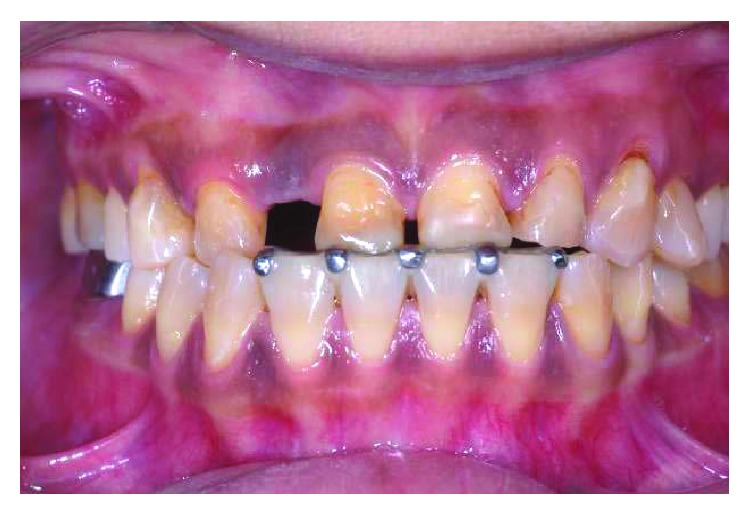
Complete reestablishment of the occlusal contacts 2 months after appliance cementation.

**Figure 10:**
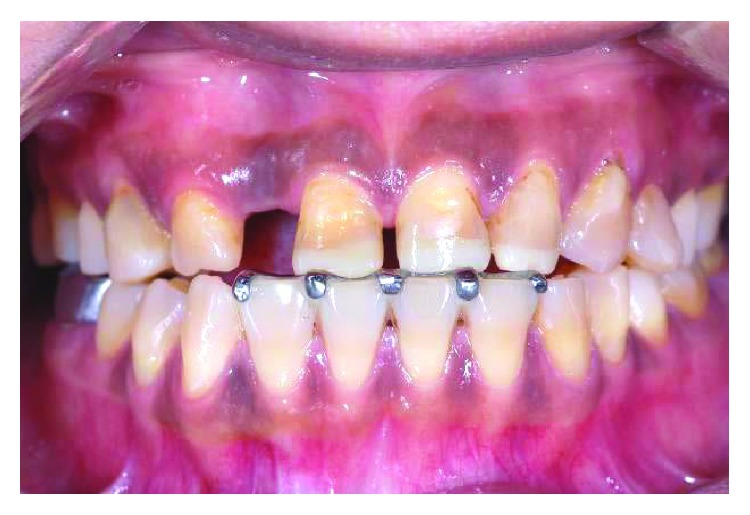
Further composite build-up on numbers 11, 21, and 22 incisal to separate all other teeth by 1.5 mm.

**Figure 11:**
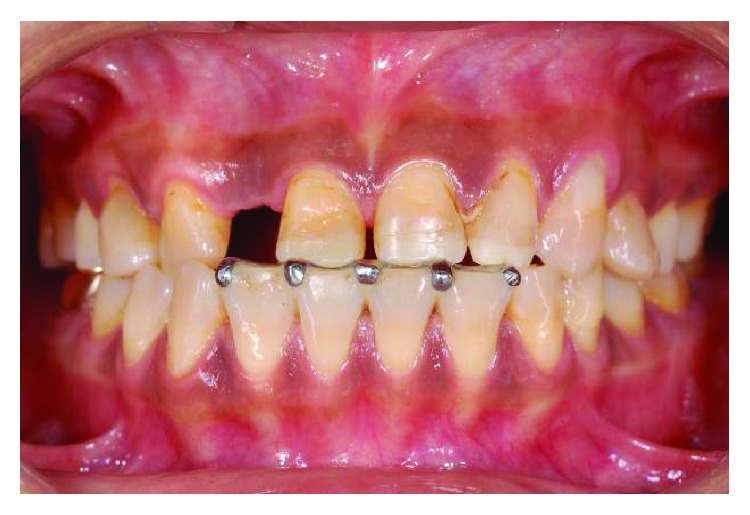
The occlusion reestablished full arch contacts over 9 months after appliance insertion, with crown heights of numbers 11 and 21 increased from 5 to 8 mm and number 22 from 5 to 7 mm.

After 2 months, the posterior teeth were found to be in occlusion (Figure 9). At this time, further “intrusion” of the upper incisors was judged to be necessary if the incisal overjet was to be changed from reverse to positive. Therefore, further composite build-up was performed on the incisal edges of 11, 21, and 22 to again create supraoccluding restorations on the lower appliance (Figure 10). The OVD was raised by another 1.5 mm which was measured between the midlabial gingival margins of 21 and 32. Complete reestablishment of all occlusal contacts was achieved after another seven months (Figure 11). Therefore, at this time, the lower supraoccluding appliance was removed by cutting off the incisal hooks and tapping off the prosthesis.

**Figure 12:**
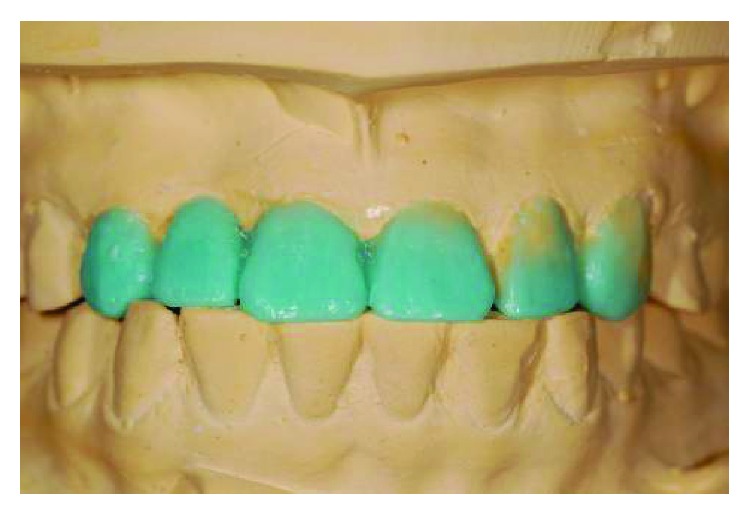
Diagnostic wax-up on number 13 to number 23 new study casts.

**Figure 13:**
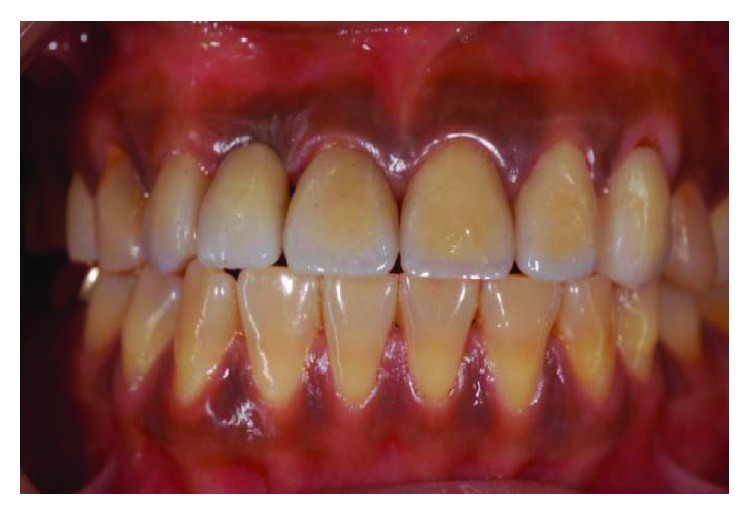
Temporary crowns in situ, which were designed to provide positive overjet. However, the crown height of numbers 11 and 21 looked short, and the tooth number 12 at 13 region did not look like a canine.

**Figure 14:**
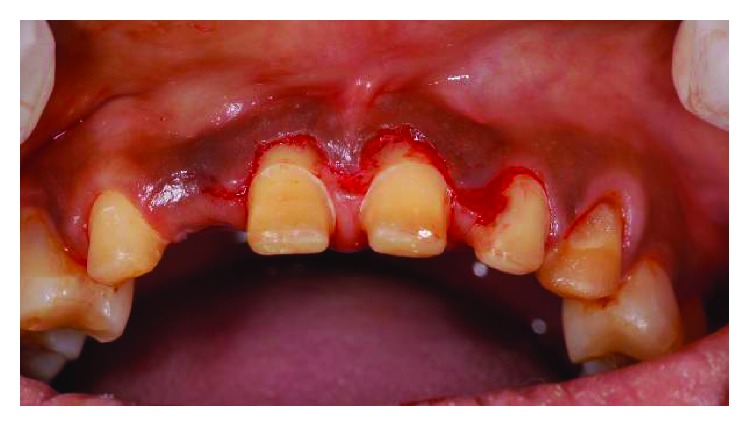
Surgical crown lengthening.

**Figure 15:**
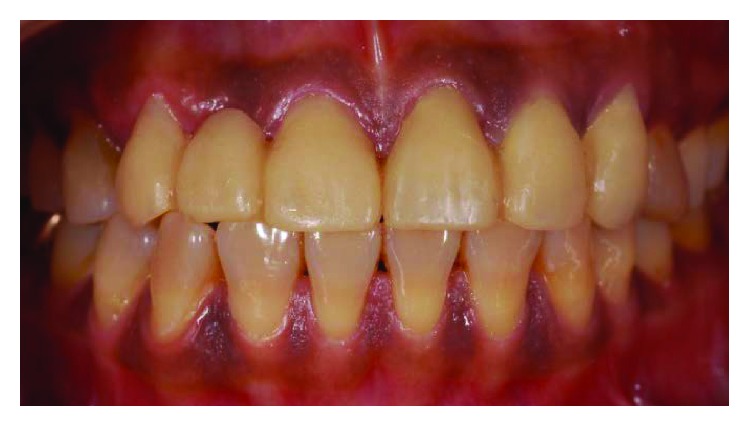
Temporary restorations numbers 13–23 in situ 1 month after crown lengthening on numbers 12, 11, and 21.

The 46 remained symptomless, and a full gold crown restoration was provided for cuspal protection. To allow tooth preparation of sufficient resistance form for the future crowns, it was decided to cement glass fibre posts on 11 and 21 with composite core build-up to restore the palatal tooth surface loss. Teeth 12 to 23 were prepared and provisional restorations made according to the diagnostic wax-up of the study casts which were taken at the new OVD and after complete reestablishment of occlusal contacts (Figure 12). With the temporary restorations in the mouth, the aesthetic outcome as well as patient's expectations were assessed (Figure 13). It was determined that lengthening the 13 to 23 incisally by 1 mm would improve the crown proportions, and crown lengthening was proposed to the patient. The soft tissue requirement was guided by a stent showing the expected final crown contour and margins. Surgical crown lengthening was performed (Figure 14) with alveolar bone being removed by approximately 1.5 mm limited to the midlabial of 12, 11, and 21. Tooth preparation margins were refined, and new temporary restorations using Protemp were made chairside using an index of the diagnostic wax-up of the expected final restorations (Figure 15).

**Figure 16:**
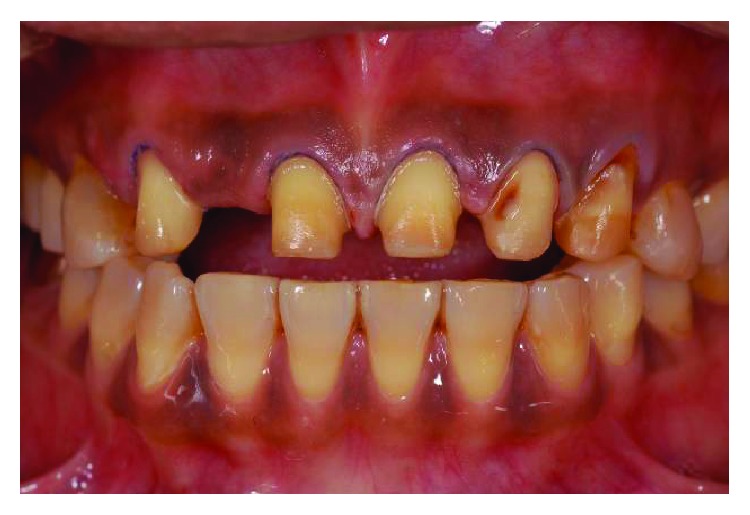
Tooth preparation on numbers 12 to 23.

**Figure 17:**
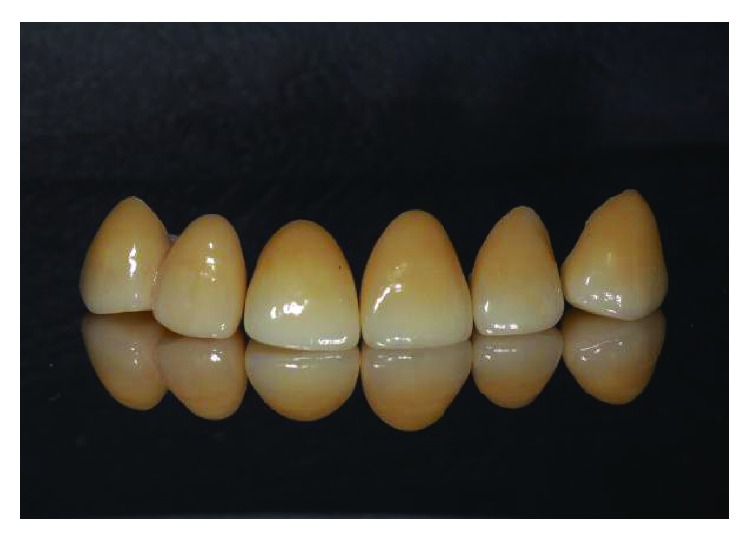
Empress crowns and bridge.

**Figure 18:**
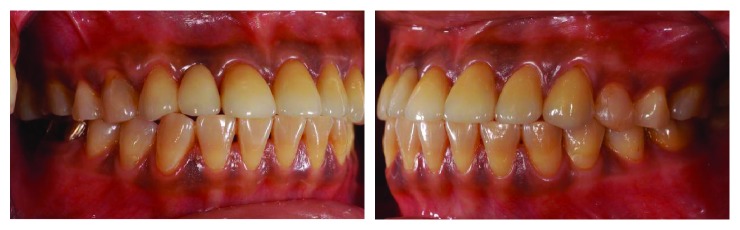
Postoperative lateral views showing successful reestablishment of the occlusion.

The intrusion of the incisors now permitted the incisal edges to be positioned with a positive overjet and overbite. A period of six months was allowed for the healing and stabilization of the soft tissues, and the patient was satisfied with the aesthetics with diagnostic restorations following review after 6 months. The form and contour of these diagnostic restorations were recorded and copied in the final restorations. Definitive restorations were provided in Empress II (Ivoclar Vivadent, Amherst, NY, USA) and cemented with an adhesive resin cement (Calibra, Dentsply, Konstanz, Germany) (Figures 16, 17, and 18).

**Figure 19:**
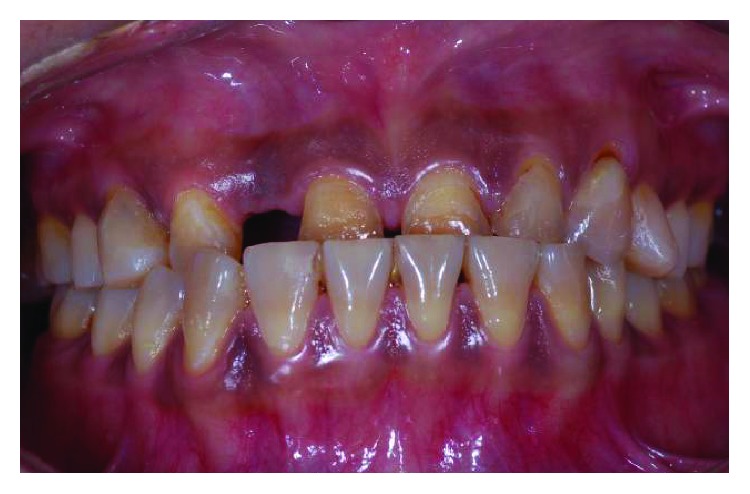
Preoperative frontal view.

**Figure 20:**
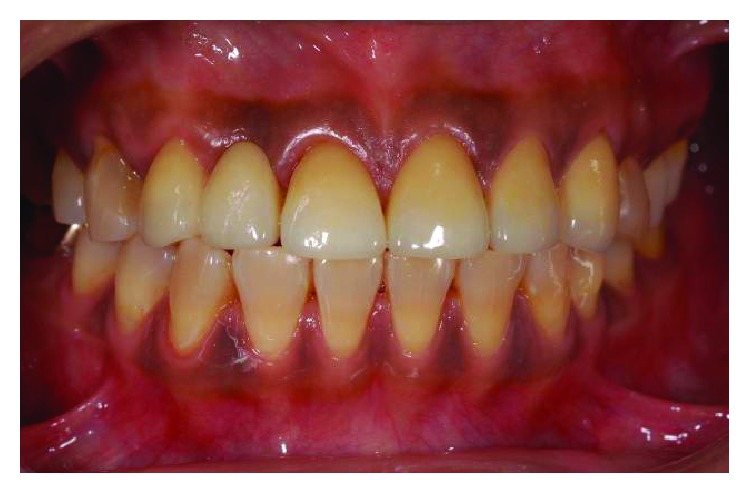
Postoperative frontal view.

**Figure 21:**
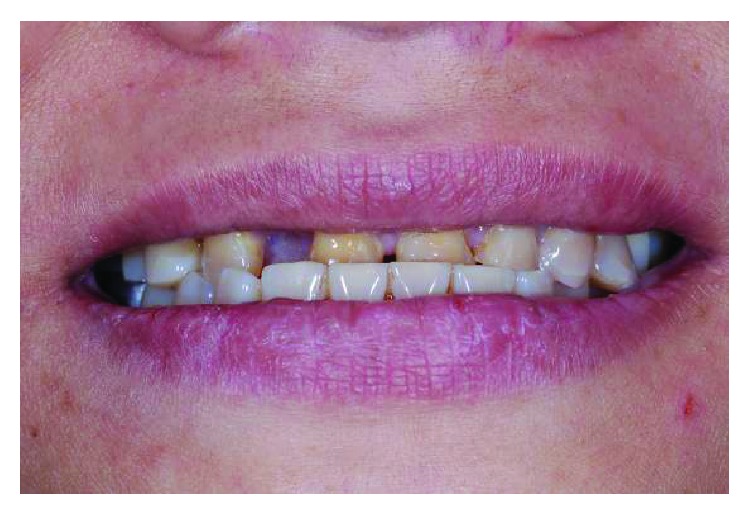
Preoperative smile line.

**Figure 22:**
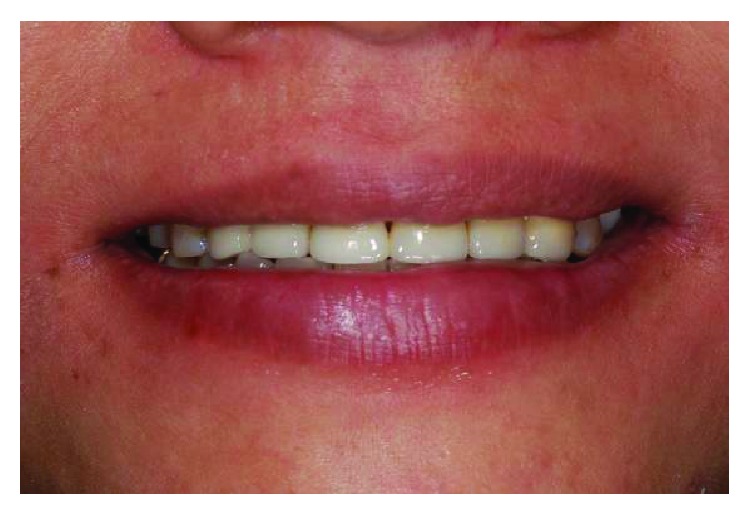
Postoperative smile line.

**Figure 23:**
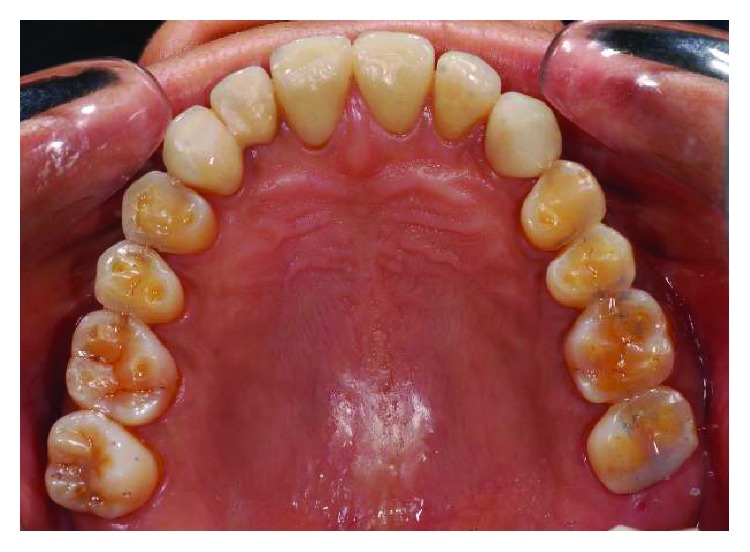
Upper occlusal view 5 years postoperatively.

**Figure 24:**
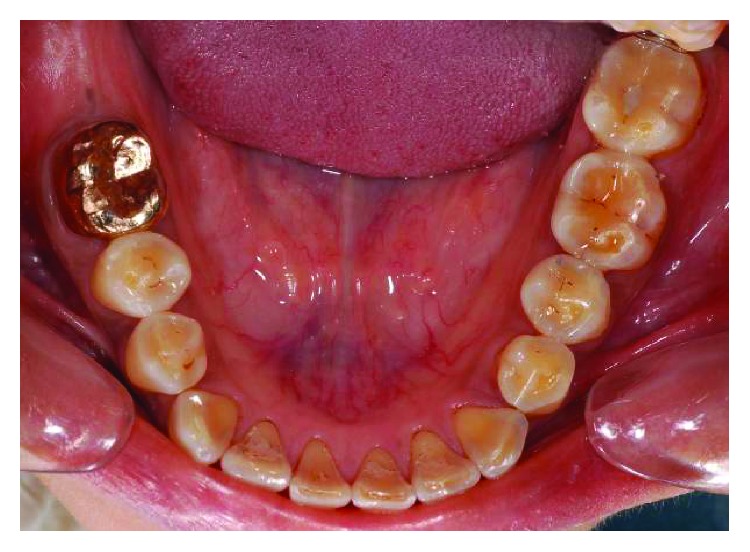
Lower occlusal view 5 years postoperatively.

**Figure 25:**
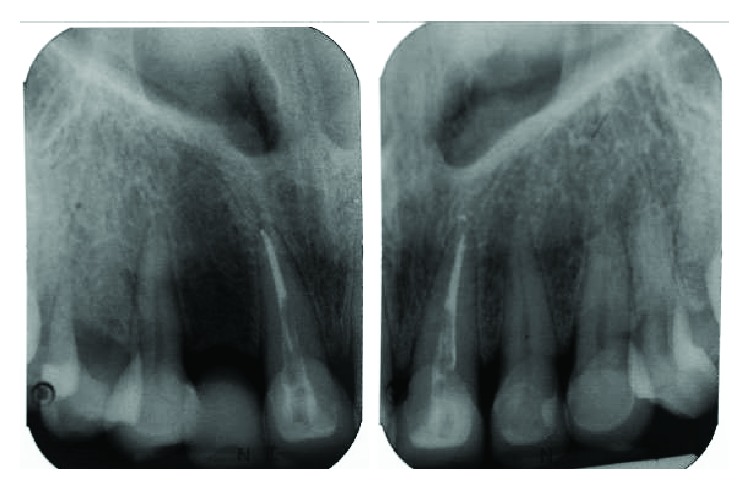
Periapical radiographs of the upper anterior teeth at review visits showing no periapical change or sign of root resorption.

The patient achieved good oral hygiene and maintained periodontal health during the follow-up period (Figures 19, 20, 21, 22, 23, and 24). No radiographic periapical change of the crowned teeth or resorption of the roots was observed at 5 years follow-up (Figure 25).

## 3. Discussion

Eating disorders and the associated regurgitation is not uncommon, and young women seem to be more at risk [11]. Because of the characteristic dental manifestations, dentists are often the first healthcare professionals to encounter patients with undiagnosed eating disorders. Although the main concern of this patient was to improve the appearance of her upper worn anterior teeth, identification and control of the etiological factors are essential before any definitive extensive restorative work [9]. This patient followed the advice to seek medical management and her gastric disturbance was controlled. There had been no more reported regurgitation or discomfort on chewing or thermal sensitivity reported. The success of the gastric reflux management may be indirectly observed by the apparent absence of observed tooth wear in the clinical photographs at the initial and subsequent treatment and review appointments.

Compliance of the patient to wear a removable appliance is also essential for treatment success, and for this reason, a fixed approach is preferred [12, 13]. Due to the reverse overjet, a novel lower bite platform was designed to disclude the posterior teeth. Because of the increased interincisal angle with the lower incisors, it may be anticipated that higher shear stresses may occur on the cement interface under occlusal loading. For this reason, incisal hooks were included to the framework to improve the resistance form. Instead of using an adhesive resin cement, a glass ionomer cement was used, and this has the advantage that the appliance could ultimately be more easily removed by tapping at the cement interface with a chisel after cutting the hooks due to the lower tensile strength.

While traditionally concern may be expressed about possible overloading of “high” crowns that may become symptomatic, studies appear to show little if any signs or symptoms being reported in supraoccluding restorations [8, 10]. There was no dental, muscle, or TMJ discomfort experienced by this patient, other than some reduced chewing efficiency initially. Follow-up periapical radiographs were taken, and there were no signs of root resorption or periapical change of the teeth concerned (Figure 25). There were no other complications, biological as well as mechanical, reported in this case during treatment and the 5-year follow-up period.

The rate of space closure to reestablish tooth contacts appears to vary among individuals and differs among different reports [5–8]. In this case, 3 mm of interocclusal space, more than enough clearance for the restorative material, was created in 9 months, within the range being reported [3–5]. The creation of the initial 1.5 mm interocclusal space occurred within 2 months, which is considered quite favorable to the literature [5–8]. However, the rate slowed down, and further intrusion (and eruption of posterior teeth) by the same amount, i.e., 1.5 mm, took a much longer period of time (7 months). The limit of the space that can be created with the Dahl concept is not known, although up to 4.7 mm has been reported [4]. Moreover, there is no mention in the literature whether vitality of the teeth, when applying this supraoccluding technique, has an effect on the tooth movement. Yet, it seems it makes no difference for the two root-treated upper central incisors.

## 4. Conclusion

This case report documents localized management of advanced erosive tooth wear in a patient with a negative overjet. A conservative and novel lower bite raising appliance was used to improve the anterior incisal relations both from a functional and aesthetic point of view. A satisfactory 5-year result is presented.
